# Using climbing to quantify motor asymmetry in children with cerebral palsy: a pilot study

**DOI:** 10.3389/fspor.2025.1541106

**Published:** 2025-10-03

**Authors:** Cecilia Monoli, Greta Simoni, Manuela Galli, Alessandro Colombo

**Affiliations:** ^1^ Department of Health & Kinesiology, University of Utah, Salt Lake City, UT, United States; ^2^ Department of Computer System, Tallinn University of Technology, Tallinn, Estonia; ^3^ Department of Electronics, Information and Bioengineering, Politecnico di Milano, Milan, Italy; ^4^ Department of Mechanical and Aerospace Engineering, Sapienza University of Rome, Rome, Italy

**Keywords:** adaptive sport, asymmetry, cerebral palsy, climbing, force sensor, motion analysis, rehabilitation

## Abstract

**Introduction:**

Adapted sports complement traditional rehabilitation for children with cerebral palsy (CP), who require continuous intervention to maintain motor function. This pilot study investigates the feasibility of using a climbing game combined with force sensors to quantify motor asymmetries in children with hemiplegic CP.

**Methods:**

Eight children with hemiplegic CP participated in climbing games for three consecutive days. Force sensors embedded in the holds measured reaction forces, while marker-less motion capture linked these forces to specific limbs. Two indices, maximum force ($F_{\max}$) and mean force ($F_{\mathrm{mean}}$), were calculated for each limb as potential proxyes for motor asymmetry. Statistical analysis using repeated measures ANOVA assessed the ability of these indices to differentiate between the more and less affected limbs.

**Results:**

The maximum force index ($F_{\max}^{\mathrm{arm}}$) successfully identified significant differences between the more affected and less affected arms in all activities (p≤0.023), with stronger results during structured tasks (p=0.002). However, neither the maximum nor the mean force indices demonstrated significant discriminatory power for the legs, likely reflecting compensatory strategies or reduced asymmetry in the lower limbs.

**Discussion:**

This pilot study supports the potential of $F_{\max}^{\mathrm{arm}}$ as a robust index to quantify upper limb motor asymmetry. Such an index could be used by therapists to track the evolution of a child’s motor abilities through a game, rather than through less pleasant clinical evaluations. The findings highlight the need for further research to validate these indices in larger cohorts, investigate their longitudinal evolution during rehabilitation, and explore correlations with clinical motor assessments.

**Conclusion:**

The results confirm the feasibility of using climbing-based force indices to detect motor asymmetries in children with hemiplegic CP. Future studies could leverage this methodology to provide quantitative feedback on the efficacy of rehabilitation interventions, fostering personalized and engaging therapeutic approaches.

## 1 Introduction

Cerebral palsy (CP), caused by a brain injury around birth or early childhood (1, 2), is a leading cause of physical disability in children worldwide. Spasticity is the most common subtype, affecting about 80% of cases (2). It results from disrupted brain signaling and is often exacerbated by movement velocity (3). In diplegic CP, it typically affects the lower limbs; in hemiplegic CP, it affects the upper extremities (4). Spasticity is marked by increased muscle tone (hypertonia), abnormal reflexes, and excessive muscle activity, leading to impairments in balance, gait, and fine motor skills (5). Over time, it contributes to secondary complications such as contractures, skeletal deformities, and increased energy expenditure (6). In addition to motor deficits, many children with CP experience altered sensation, intellectual disabilities, behavioral and communication challenges, seizures, and chronic pain, all of which can significantly limit participation and reduce quality of life (2, 7).

Although the brain injury underlying CP is non-progressive, its clinical manifestations can worsen over time as the central nervous system matures (8). Continuous physical activity is essential to maintain motor function, prevent secondary complications, and promote independence. However, traditional rehabilitation often requires intensive clinical sessions or hospitalization, which can disrupt family routines and limit opportunities for social engagement. As a result, there is growing interest in integrating complementary forms of physical activity with clinical rehabilitation, such as adapted sports, that are enjoyable and easily integrated into daily life (9–11).

Among adapted sports, climbing has shown benefits for both upper and lower limb mobility (12, 13), hand grip strength, postural control, coordination (14, 15), and spasticity reduction (15). Furthermore, it is associated with positive psychosocial effects (16). Concurrently, recent technological advances in motion analysis are being applied to climbing, enabling quantitative assessment with minimal interference to the participant’s experience (17–21).

Building on this, this pilot study explores whether sensor-based climbing technologies can be used to assess motor function in children with cerebral palsy. Specifically, this study does not aim to evaluate the therapeutic efficacy of climbing, nor to address spasticity directly. Rather, it investigates whether force sensors embedded in a climbing wall can serve as a tool to assess motor asymmetry in children with hemiplegic CP during typical climbing exercises. Climbing requires dynamic bilateral coordination and weight-bearing through all limbs, providing a natural context in which asymmetries, particularly in hemiplegia, may emerge as measurable differences in the forces applied to climbing holds. We hypothesize that asymmetries in upper and lower limb motor control will be reflected in the distribution and magnitude of forces applied to climbing holds. Additionally, for a therapeutic activity to complement standard rehabilitation, it must be both effective and engaging. Standardized motion assessments typically involve repetitive, constrained tasks that may not capture natural movement or hold a child’s attention. To address this, we designed both structured (with constraints on the allowed movements) and unstructured (freeform, with little or no movement constraints) climbing games to evaluate the proposed approach in a playful and ecologically valid context.

We report results from a group of children with mild to moderate hemiplegic CP engaged in structured and unstructured climbing tasks. We propose novel indices derived from force sensor data and assess their ability to quantify asymmetries between the more and the less affected limbs. Although motor asymmetry in children with hemiplegic CP is clinically recognized, an objective and task-specific quantification during functional activities such as climbing could, after longitudinal validation, provide therapists with quantitative indicators to better tailor rehabilitation strategies. By leveraging the engaging nature of climbing, our long-term goal is to provide clinician a complementary and ecologically valid method of objective motor assessment, to complement conventional clinical assessments.

## 2 Methods

### 2.1 Participants

The study involved eight children with CP (1 Female and 7 Males), aged between 7 and 11 years. Four participants had left-sided hemiplegia, and four had right-sided hemiplegia, with all children classified as Class I or II in the Gross Motor Function Classification System (22). Inclusion criteria required participants to have hemiplegia resulting from a perinatal stroke, no additional comorbidities, and written medical approval from their physicians.

The children were recruited among the participants of a one-week intensive rehabilitation program called *Fight Camp*, organized by the *FightTheStroke* foundation. Written informed consent was obtained from all parents or legal guardians prior to participation. The study was approved by the Ethics Committee of Politecnico di Milano (Session 24/2021) and was conducted in accordance with the ethical principles outlined in the Declaration of Helsinki.

### 2.2 Adapted climbing wall and force sensors

**Figure 1:**
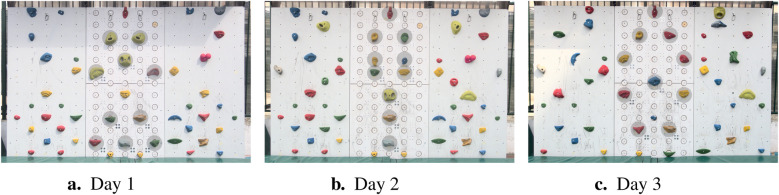
Configurations of the climbing wall during the three measurement days. Sensorized holds are indicated by gray circles.

We used an adapted bouldering wall measuring 3.66 m in width and 2.44 m in height (Figure 1). The wall featured hold attachments spaced 23 cm horizontally and 18 cm vertically. To ensure safety, the wall was equipped with fall-protection mattresses compliant with EN12572-2 standards. The wall was suitable for climbing without rope and harness but also allowed the use of a manually operated rope and harness to support climbers as needed.

**Figure 2:**
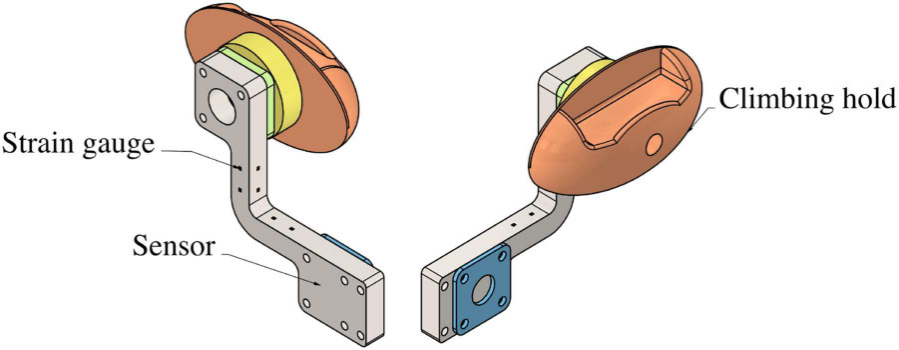
Schematics of the force sensors embedded in the climbing wall. The sensing component (in grey) is mounted behind the wall. A wooden disk 8cm in diameter (yellow) slightly protrudes from the front of the wall and provides a sufficient surface to securely attach a standard climbing hold (17).

The climbing wall was divided into three sections, with the central portion designed to be compatible with the integration of force sensors. Ten customized triaxial force sensors (Figure 2), developed by Politecnico di Milano, were used in this investigation. Each sensor was mounted behind the wall and appeared to the climbers as a circular disk matching the wall’s color and texture for seamless integration. The sensors securely supported standard artificial climbing holds.

The sensors were designed to record the magnitude and direction of forces exerted on the climbing holds, measuring the three components of the force vector irrespective of the exact point of force application, as they were designed to reject moments. Force signals were sampled at 80 Hz and synchronized across all sensors. Signals were wirelessly transmitted to a tablet, where a custom application enabled real-time visualization, basic processing, and data export for further analysis. Force signals were subsequently processed using MATLAB. A low-pass filter with a 1 Hz bandwidth was applied using an infinite impulse response (IIR) zero-phase filter (MATLAB *lowpass* function, 0.95 steepness) to reduce noise and ensure signal clarity. For detailed technical information about the sensor design, their operational principles, and the mobile application, please refer to (17).

### 2.3 Experimental protocol

Participants engaged in a seven-day intensive bimanual rehabilitation program guided by physical and occupational therapists, alongside other specialists. Each child spent 30 min daily on activities involving the climbing wall. The first four days served as a familiarization phase, during which children became accustomed to climbing. Measurements were collected during the final three days, with each child performing two climbing exercises per day, referred to as “*repetitions*.” During these exercises, children climbed the sensorized section of the wall while wearing a harness connected to a safety rope. They were free to choose their movements and positions, with no specific instructions provided. In cases requiring assistance, instructors held the rope for additional guidance.

While the purpose of the study was to determine whether forces exerted on the holds could detect motor asymmetries, the climbing exercises were collaboratively designed by physical and occupational therapists to accommodate the children’s anthropometric parameters and motor abilities. Hold placement, spacing, shape, and orientation were optimized to facilitate specific movements, such as elbow extension, hand pronation-supination, grasping, and finger extension. The climbing routes were intentionally symmetrical, ensuring that both left-hemiplegic and right-hemiplegic children faced equivalent challenges.

#### 2.3.1 Day 1 and Day 3: unstructured, goal-oriented climbing

**Figure 3:**
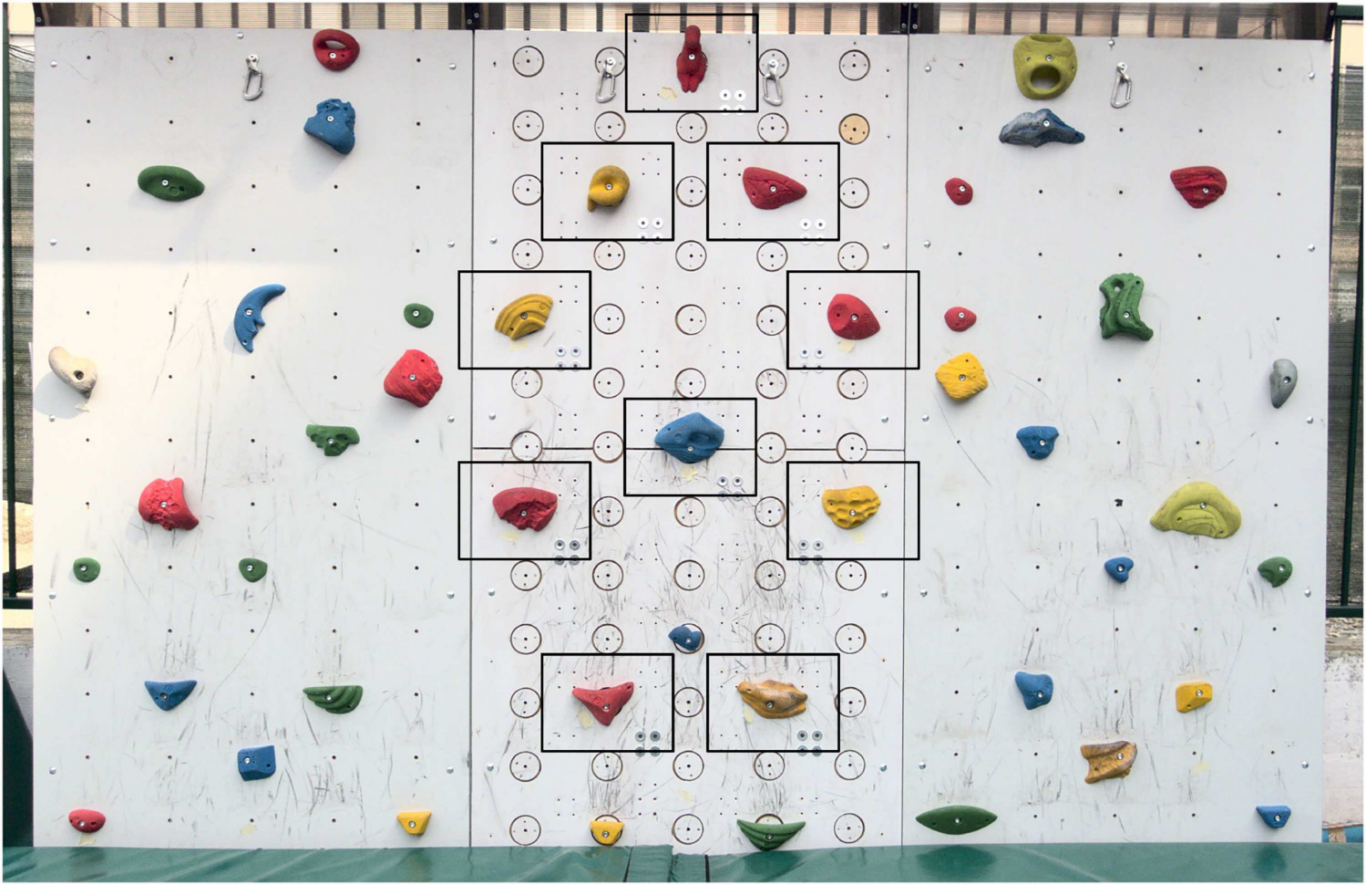
Non-overlappint boxes used to associate the limb to the corresponding recorded force. The boxes are centered on each of the 10 sensorised holds used on the climbing wall used in Day 3.

On Days 1 (Figure 1a) and 3 (Figure 1c), children participated in a playful, unstructured climbing exercise. The children climbed the central panel aiming at reaching the top hold shaped like a dinosaur. While the goal was fixed, children were free to choose their climbing movements. On Day 1 (Figure 1a), the holds were arranged to promote wrist extension and fine motor skills. On Day 3 (Figure 1c), the arrangement encouraged maximum elbow extension of the more affected arm.

#### 2.3.2 Day 2: structured game, “Pull the hold”

On Day 2 (Figure 1b), children participated in a structured climbing game called *Pull the hold*. The sensorized panel featured three pairs of holds of equal height in the upper half and three large footholds in the lower half. The goal was to reach the dinosaur-shaped top hold, but the climbing path required sequential placement of the hands on paired holds. When childrens’ hands were at the same height, they were asked to pull pairs of holds as hard as possible. Instructors encouraged children to place their feet on footholds that promoted a frontal position and symmetrical arm use. Instructors provided real-time feedback on the maximum force exerted to motivate the children. This gamified approach mirrored standard laboratory dynamometer tests (23), integrating assessment with an engaging, child-friendly activity.

### 2.4 Data processing

To reduce noise, a force threshold of $f_{\text{min}}=1\;\text{kgf}$ was applied. Let F represent the time series of force data recorded by a sensor during a climbing task, with $F(t)\in R^{3}$ denoting a sample at time t. A *force event* was defined as any time interval $\left[t_{a},t_{b}\right]$ in which $\|F(t)\|>f_{\text{min}}$ for all $t\in [t_{a},t_{b}]$, where ‖⋅‖ denotes the Euclidean norm.

The climbing wall and its force sensors cannot independently identify which limb was in contact with a given hold at a given time but recorded solely the forces exerted on each hold over time. To associate the force events with the specific limbs of the climbers, it was necessary to integrate force sensor readings with an external motion capture system. All climbing exercises were recorded using a front-facing camera (Sony A6000, 30 fps). The video recordings were manually synchronized with the force time series by aligning the first contact of a limb with a hold (observed in the video) to the corresponding initial force event. The optimal time delay between video frames and force data was determined using a least-squares minimization of the time differences between paired instances.

The video recordings were processed with OpenPose, a markerless video analysis software that analyzes each frame independently to detect all visible subjects (2). Using the BODY_25_model, OpenPose extracted the two-dimensional pixel coordinates of 25 anatomical keypoints in each frame of the video, four of which corresponded to the hands and feet. These trajectories were used to determine which limb was touching a hold when a nonzero force was detected. A limb was therefore assigned to a force event if, during the event, the limb’s keypoint remained within a bounding box around the corresponding hold, as shown in Figure 3. Force events involving multiple limbs touching the same hold simultaneously were excluded from the dataset to ensure unambiguous assignment. To improve reliability, all detected limb contacts were manually verified frame-by-frame based on the visual output of OpenPose and bounding box overlaps with the holds. This ensured correct temporal alignment between video and force data.

This process resulted in a set of paired data points (force event,limb). Each time series described the evolution of a force vector with a magnitude greater than $f_{\text{min}}$, exerted by the more or less affected limb of the child. By concatenating all force events associated with the same limb, four time-series were generated for each participant: (1) $F_{\text{arm, l}}$, force time series for the less affected arm; (2) $F_{\text{arm, m}}$, force time series for the more affected arm; (3) $F_{\text{leg, l}}$, force time series for the less affected leg; (4) $F_{\text{leg, m}}$, force time series for the more affected leg. This procedure was repeated for all participants, across all measurement days and repetitions, resulting in a total of 48 quadruplets (8 participants × 3 days × 2 repetitions per day).

### 2.5 Force indices

Four time-series for each child, repetition, and measurement day were obtained, representing the force vectors exerted by each of the four limbs during climbing. Based on these time series, we defined two indices to assess motor asymmetry.

The first index, $F_{\text{max}}^{i}$ (Equation 1), represents the maximum force magnitude exerted by a given limb i (i∈{arm, l; arm, m; leg, l; leg, m}), expressed as a percentage of the child’s body weight:

(1) $$F_{\text{max}}^{i}=\max_{t\in T_{i}}\left(\frac{100\cdot \|F^{i}(t)\|}{w}\right),$$

where $T_{i}$ is the set of time samples in the time series for limb i, $\left\|F^{i}(t)\right\|$ is the Euclidean norm of the force vector at time t, and w is the child’s body weight, measured using a standard scale before the exercise. This index captures the peak force exerted by a limb during the climbing task, normalized by body weight.

The second index, $F_{\text{mean}}^{i}$ (Equation 2), measures the average force magnitude exerted by limb i, also normalized by the child’s body weight:

(2) $$F_{\text{mean}}^{i}=\frac{1}{N}\sum_{t\in T_{i}}\left(\frac{100\cdot \|F^{i}(t)\|}{w}\right),$$

where N is the total number of time samples in $T_{i}$. This index reflects the overall force exerted by a limb during the entire climbing task, averaged over time and expressed relative to the child’s weight.

Both indices were computed for each limb, across all eight children, for every repetition (two per day) and each measurement day (three days total). These indices were evaluated as potential metrics to detect motor asymmetry by comparing the performance of the more and less affected limbs.

### 2.6 Statistical analysis

Statistical analysis was conducted using the Statistical Package for the Social Sciences (SPSS, IBM). The indices $F_{\text{mean}}$ and $F_{\text{max}}$ were first tested for normality using both the Kolmogorov-Smirnov and Shapiro-Wilk tests.

The primary objective of the analysis was to evaluate whether the forces recorded during the climbing activities could effectively identify motor asymmetries in children with CP. To this end, repeated-measures Analysis of Variance (ANOVA) was employed. The results from Day 2 were analyzed separately from those of Days 1 and 3 due to the differing nature of the activities (structured vs. unstructured climbing tasks).

For Days 1 and 3, the indices were analyzed separately for arms and legs using a three-way repeated-measures ANOVA. The within-subject factors included the limb (more affected or less affected), the measurement day (Day 1 or Day 3), and the exercise repetition (two repetitions per day). On Day 2, the indices $F_{\text{mean}}$ and $F_{\text{max}}$ were calculated for the arms only and analyzed using a two-way repeated-measures ANOVA. The within-subject factors were the limb (more affected or less affected) and the exercise repetition (two repetitions per day).

## 3 Results

Both the goal-oriented, unstructured climbing exercises performed on Days 1 and 3, and the more structured game of *Pull the hold* performed on Day 3, were designed to evaluate whether the proposed indices of maximum and average force could effectively discriminate between the more and less affected limbs in children with CP.

**Figure 4:**
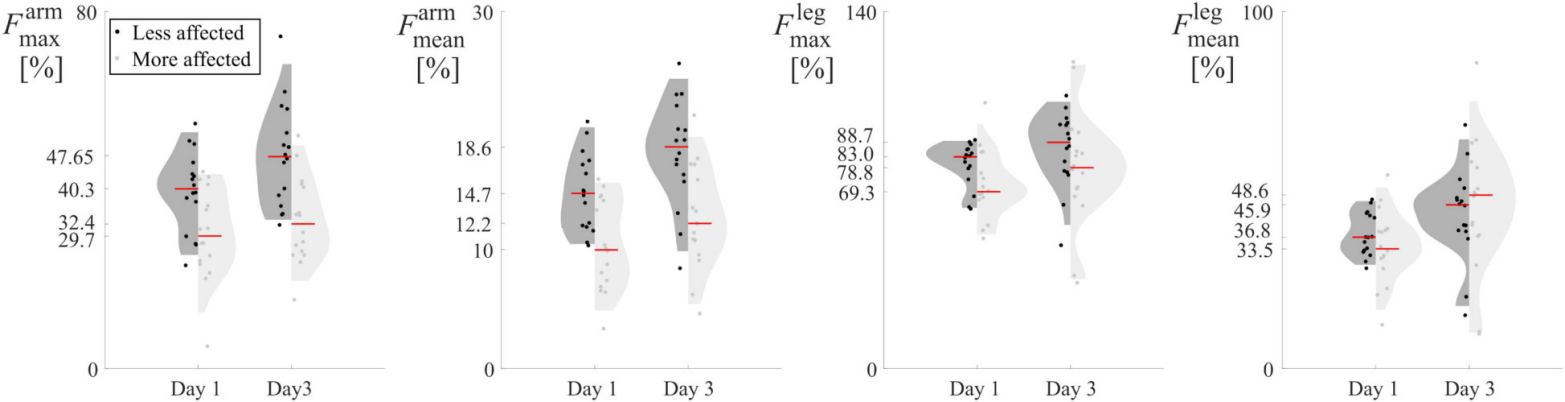
Violin plots of the force indices measured during Day 1 and Day 3 unstructured climbing exercises, for both upper ($F_{\max}^{\mathrm{arm},l}$, $F_{\max}^{\mathrm{arm},m}$), ($F_{\mathrm{mean}}^{\mathrm{arm},l}$, $F_{\mathrm{mean}}^{\mathrm{arm},m}$), and lower limbs ($F_{\mathrm{mean}}^{\mathrm{leg},l}$, $F_{\mathrm{mean}}^{\mathrm{leg},m}$), and ($F_{\mathrm{mean}}^{\mathrm{leg},l}$, $F_{\mathrm{mean}}^{\mathrm{leg},m}$). More and less affected limbs are reported in gray and black, respectively. The red lines highlight the median of each distribution, whose values are reported on the *y*-axis.

**Figure 5:**
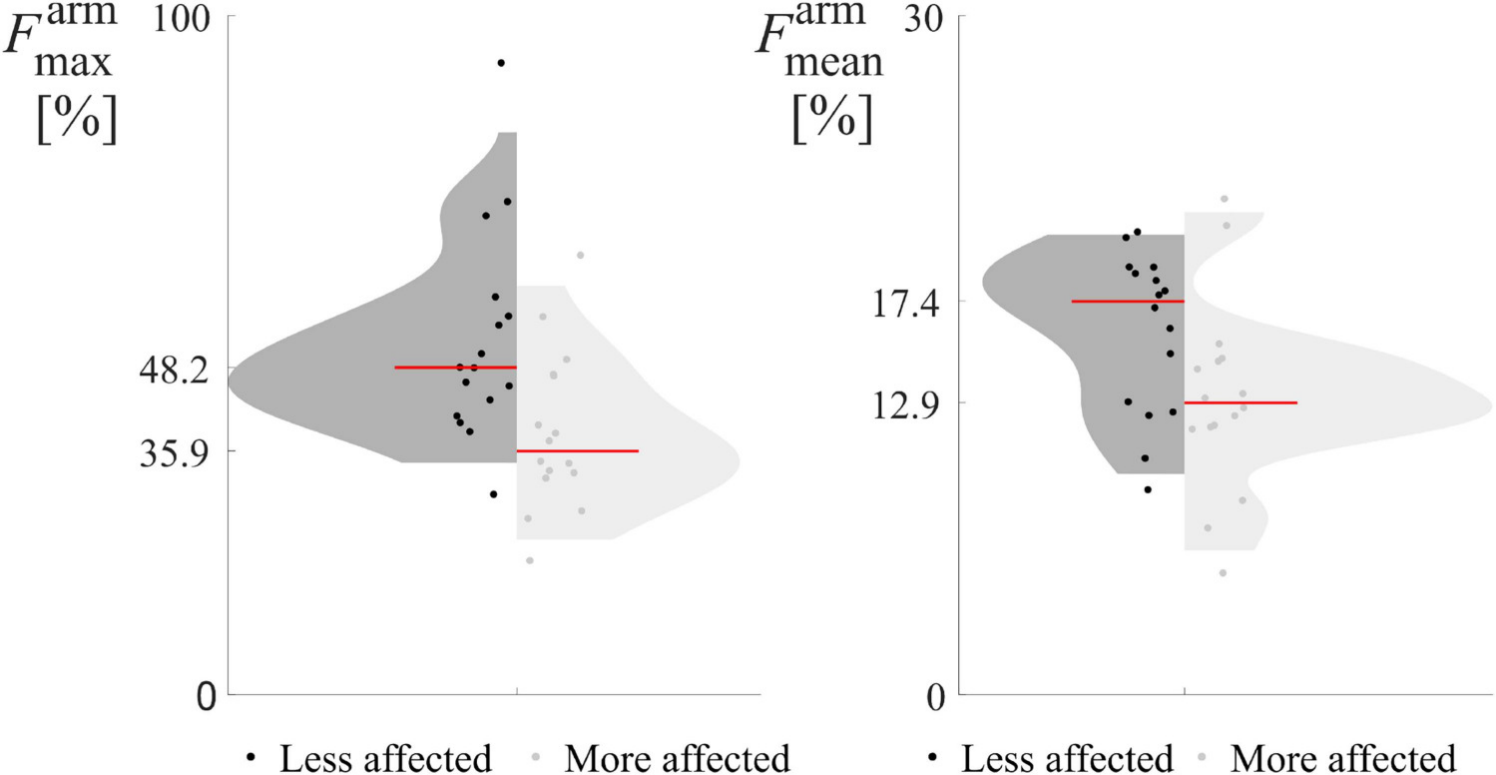
Violin plots of the force indices measured during Day 2, *Pull the hold*, exercise, for the upper limbs ($F_{\max}^{\mathrm{arm},l}$, $F_{\max}^{\mathrm{arm},m}$), ($F_{\mathrm{mean}}^{\mathrm{arm},l}$, $F_{\mathrm{mean}}^{\mathrm{arm},m}$), the more and less affected arms are reported in gray and black, respectively. The red lines highlight the median of each distribution, whose values are reported on the *y*-axis.

Results per each subject and repetition are reported in violin plots in Figures 4, 5, to illustrate the distribution of force indices. The less affected limb is reported on the left in black, and the more affected limb on the right in light gray. Each plot includes a red line denoting the median, with values displayed on the vertical axis. F-values and p-values outcomes of the repeated-measures ANOVA are reported in Tables 1, 2, with dashes indicating no statistical significance.

**Table 1:** ANOVA test results for Days 1 and 3.

Model factors	$F_{\max}^{\mathrm{arm}}$		$F_{\mathrm{mean}}^{\mathrm{arm}}$		$F_{\max}^{\mathrm{leg}}$		$F_{\mathrm{mean}}^{\mathrm{leg}}$	
	F(1,7)	p	F(1,7)	p	F(1,7)	p	F(1,7)	p
Limb	8,379	0.023	8,471	0.020	–	–	–	–
Day	7,056	0.033	32,826	<0.001	–	–	7,485	0.029
Repetition	–	–	–	–	–	–	–	–

The dash indicates that no significant differences were found.

**Table 2:** ANOVA test results for Day 2.

Model factors	$F_{\max}^{\mathrm{arm}}$		$F_{\mathrm{mean}}^{\mathrm{arm}}$	
	F(1,7)	p	F(1,7)	p
Limb	21,330	0.002	–	–
Repetition	–	–	–	–

The dash indicates that no significant statistics were found.

### 3.1 Day 1 and Day 3

Both maximum and average force indices during the unstructured climbing games of Day 1 and Day 3, reported higher median values for the less affected limbs than those of the more affected limbs (Figure 4).

To evaluate the statistical significance of these differences, the three-way repeated measures ANOVA was performed, with within-subject factors limb (more affected or less affected), day (Day 1 or Day 3), and repetition (two repetitions per day). The limb factor was significant for both maximum (p=0.023) and mean (p=0.020) force indices, suggesting that both indices successfully distinguished between the more and less affected arms. However, for $F_{\mathrm{mean}}^{\mathrm{arm}}$, this distinction depended on day and repetition due to significant interactions between the three factors (F(1,7)=6.181,p=0.042).

Conversely, neither lower limbs index effectively discriminated between the more and less affected legs. The Day factor was significant for the mean force index, reflecting differences in the movement strategies employed to complete the tasks on Days 1 and 3 (p=0.029). The Repetition factor, however, was not significant, supporting the reliability of the measures across repeated trials. Additionally, no other interaction effects were significant for both upper and lower limb indices.

### 3.2 Day 2

The structured climbing exercise on Day 2, *Pull the hold*, focused exclusively on upper limb performance (Figure 5). This activity aimed to determine whether the force indices could detect motor asymmetries when the task resembled a dynamometric test. Since the hold placing included three large footholds in the lower half of the wall to promote symmetric arm use, the contributions of individual legs could not be distinguished and the leg force indices were not calculated for this day.

Similarly to what was seen in Days 1 and 3, the median indices (red lines in Figure 5) for the less affected arm were consistently higher, indicating greater maximum or average force compared to the more affected arm.

The two-way repeated measures ANOVA evaluated statistical significance (Table 2), with the within-subject factors being limb (more affected or less affected) and repetition (two per day). The maximum force index ($F_{\max}^{\mathrm{arm}}$) successfully discriminated between the more and less affected arms (p=0.002). However, the average force index ($F_{\mathrm{mean}}^{\mathrm{arm}}$) did not exhibit a significant difference between the limbs. Additionally, neither the repetition factor nor the interaction effects were significant, indicating consistent performance across repetitions and no confounding effects.

## 4 Discussion

This pilot study investigated whether climbing-based force measurements can detect motor asymmetries in children with hemiplegic CP. Eight participants with CP-related hemiplegia participated in climbing activities across across three sessions involving both structured and unstructured climbing activities, where we examined the maximum and average forces exerted by each limb to identify asymmetry.

The maximum force index for the arms ($F_{\max}^{\mathrm{arm}}$) consistently differentiated between the more and the less affects arms across all days (p=0.023), with the strongest effect observed during the structures task on Day 2 (p=0.020). This structured setting, where children pulled on identical paired holds, likely reduced task variability and amplified the motor asymmetry signal. The observed day effect for $F_{\max}^{\mathrm{arm}}$ suggests that task design significantly influences performance and measurement sensitivity. These findings support the robustness of $F_{\max}^{\mathrm{arm}}$ as a potential clinical marker of upper-limb asymmetry in children with hemiplegic CP, even during free climbing.

In contrast, the average force index ($F_{\mathrm{mean}}^{\mathrm{arm}}$) showed inconsistent results. While it reached significance on Days 1 and 3, it did not differentiate between arms during the structured task on Day 2. Moreover, factor interactions on Days 1 and 3 complicate its interpretation and suggest limited robustness. As such, caution is warranted when using $F_{\mathrm{mean}}^{\mathrm{arm}}$ as a standalone indicator of asymmetry.

Neither the maximum nor average leg force indices ($F_{\max}^{\mathrm{leg}}$ and $F_{\mathrm{mean}}^{\mathrm{leg}}$) significantly distinguished between limbs. This may reflect both biomechanical and behavioral factors. Hemiplegia often presents more prominently in the upper limbs, and children may unconsciously adopt compensatory weight-distribution strategies that mask asymmetries in the lower limbs during climbing. Future task designs targeting unilateral leg engagement, or the integration of limb-specific wearable sensors, may help overcome this limitation.

The findings of this study support the hypothesis that climbing activities, coupled with sensorized force measurements, could be used as a tool for assessing motor asymmetry in children with hemiplegic CP. While the proposed climbing-based approach offers a more engaging, child-friendly context, the absence of standard clinical mobility assessments, such as handheld dynamometry, Gross Motor Function Measure, Melbourne Assessment or the Assisting Hand Assessment, limits the ability to contextualize the force-based indices in relation to motor function scales. Including such assessments and test–retest reliability or intra-session consistency analysis, would establish the robustness of the indices and validate their clinical relevance and utility. Additionally, the climbing tasks were not explicitly designed to isolate specific movement patterns, which may reduce reproducibility across studies or populations. Future designs should incorporate targeted challenges (e.g., single-limb tasks) and include wearable sensors to capture limb-specific contributions more accurately, especially in the lower limbs.

Despite the consistent discrimination of upper limb asymmetries by $F_{\max}^{\mathrm{arm}}$ is promising across both structured and unstructured activities, these results should be interpreted in the context of a small sample size and pilot design. This work is not powered to detect small-to-moderate effects with high confidence. As such, nonsignificant findings must be interpreted with caution, as they may reflect insufficient power rather than true absence of effect (i.e., potential Type II errors). These limitations highlight the need for larger, adequately powered studies to confirm the observed trends and further assess the sensitivity of these indices. The participant group was limited to children with hemiplegic CP classified at GMFCS levels I–II. While this population is a logical first step for a feasibility study, the findings may not generalize to children with more severe motor impairments or other CP subtypes (e.g., diplegic or dyskinetic CP). Future work should evaluate the feasibility and adaptability of this method in broader populations and assess whether similar force-based indices remain valid across different motor profiles. From a methodological standpoint, limb contact was determined using OpenPose and bounding boxes, a practical solution for this pilot setting. However, we did not perform a formal validation of OpenPose accuracy. While visual inspection confirmed appropriate limb labeling, future studies should systematically evaluate tracking precision against a ground-truth reference to quantify reliability and minimize bias due to potential misclassifications.

Although subject to these limitations, this study provides preliminary evidence that climbing-based assessments, particularly those incorporating $F_{\max}^{\mathrm{leg}}$ may serve as engaging, objective tools to quantify motor asymmetry in pediatric populations. Longitudinal studies are needed to determine whether these indices are sensitive to therapeutic change and suitable for monitoring rehabilitation progress.

Finally, integrating such assessments into sensorized climbing environments like the ACCEPT wall offer exciting opportunities for combining and integrating therapy and evaluation in a playful, real-world context. By providing real-time objective feedback and supporting individualized rehabilitation, these systems could support the design of personalized rehabilitation protocols. The inclusion of such activities in regular therapy programs could encourage physical activity in a fun, engaging, and measurable way, bridging the gap between clinical environments and real-world functional tasks.

## 5 Conclusions

This climbing pilot study bridges the conceptual space between clinical assessment and adapted physical activity, providing a novel method to quantify motor asymmetry while promoting autonomous, inclusive participation. The study demonstrates the feasibility of using climbing-based force indices to detect motor asymmetries in children with hemiplegic cerebral palsy during both structured and unstructured climbing activities on a sensorized climbing wall. The maximum force exerted by the upper limbs ($F_{\max}^{\mathrm{arm}}$) consistently distinguished between the more and less affected arms across various climbing tasks, including free-form activities. This highlights its robustness as a measure for assessing motor asymmetry in dynamic, real-world scenarios. In contrast, the average force index ($F_{\mathrm{mean}}^{\mathrm{arm}}$) showed less consistency, and indices for the lower limbs were not sensitive to asymmetries, likely reflecting task design limitations and the clinical characteristics of hemiplegia.

Beyond demonstrating feasibility, these findings suggest that sensorized climbing walls such as the ACCEPT wall could eventually serve as practical tools to complement traditional clinical assessments. By offering quantitative and task-specific measures of asymmetry in a playful, non-clinical environment, climbing tasks may help therapists obtain objective feedback to guide and personalize interventions. While such measures are not intended to replace formal medical evaluations, they hold promise for providing more frequent, accessible, and engaging monitoring of motor performance in everyday practice. This exploratory work therefore lays the foundation for longitudinal studies aimed at validating climbing-based force indices as clinically meaningful biomarkers of motor asymmetry. If confirmed, these measures could ultimately inform tailored rehabilitation strategies, strengthening the role of adapted physical activity not only in participation and inclusion, but also in shaping future evidence-based therapeutic approaches.

## Data Availability

The raw data supporting the conclusions of this article will be made available by the authors, without undue reservation.
